# Application of tomosynthesis for vertebral compression fracture diagnosis and bone healing assessment in fracture liaison services

**DOI:** 10.3389/fmed.2022.910130

**Published:** 2022-09-16

**Authors:** Hsuan-Yu Chen, Tuoh Wu, Sheng-Pin Tseng, Chia-Yu Lin, Chih-Wei Chen, Tze-Hong Wong, Yuh-Fen Wei, Ya-Fang Chen

**Affiliations:** ^1^Department of Orthopedics, National Taiwan University College of Medicine and National Taiwan University Hospital, Taipei, Taiwan; ^2^Department of Orthopedics, National Taiwan University Hsin-Chu Hospital, Hsinchu, Taiwan; ^3^Department of Biological Science and Technology, National Yang Ming Chiao Tung University, Hsinchu, Taiwan; ^4^Health Physics Division, Institute of Nuclear Energy Research, Atomic Energy Council, Taoyuan, Taiwan; ^5^Department of Medical Imaging, National Taiwan University Hsin-Chu Hospital, Hsinchu, Taiwan; ^6^Department of Medical Imaging, National Taiwan University College of Medicine and National Taiwan University Hospital, Taipei, Taiwan

**Keywords:** tomosynthesis, vertebral compression fractures, osteoporosis, precision, fracture liaison service, cost-effective

## Abstract

Early identification of vertebral compression fractures (VCFs) is crucial for successful secondary fracture prevention. Tomosynthesis, a low-dose tomographic imaging technique, may facilitate the evaluation and long-term follow-up of VCFs in patients with osteoporosis. Herein, we compared the performances of plain radiography and tomosynthesis for VCF diagnosis and healing assessment in patients enrolled in fracture liaison services in our hospital. Forty-nine patients with new VCFs at the T10–L5 levels were prospectively recruited between August 2018 and May 2020; all patients underwent thoracolumbar plain radiography and tomosynthesis. We evaluated the accuracy of the VCF diagnosis, image quality, and VCFs healing process. Tomosynthesis identified 90 levels of VCF in 49 patients, while plain radiography revealed only 87.8% (79/90) of them. There were 44.9% (22/49) patients with neglected chronic VCFs as seen on tomosynthesis. Tomosynthesis images had improved VCF diagnostic accuracy up to 12.2% and showed significantly more anatomic details than plain radiography. For diagnosis of VCFs, the performance of plain radiographs was poorer than that of tomosynthesis images (plain radiographs: sensitivity 84%, specificity 93.5%, false positive rate 6.5%, and false negative rate 16%; tomosynthesis: sensitivity 93.2%, specificity 100%, false positive rate 0%, and false negative 6.8%), using magnetic resonance imaging (MRI) as gold standard. The Kappa coefficient between Tomosynthesis and MRI is 0.956 while between radiography and MRI is 0.704. Tomosynthesis showed significantly more anatomic details than plain radiography and all the examiners revealed a clear preference for tomosynthesis. Tomosynthesis scored 3.3 times higher on the fracture healing assessment at the 3-month follow-up than plain radiographs. Tomosynthesis is a promising tool for VCF screening and diagnosis in patients with osteoporosis and for monitoring fracture healing status at a low radiation dose and cost.

## Introduction

Vertebral compression fractures (VCFs), which occur due to reduced bone strength, particularly in the trabecular bone, are the hallmark of osteoporosis, and they exert a substantial risk for subsequent fractures, disability, and morbidity (1). Two-thirds of VCFs are clinically silent, so routine thoracolumbar spine radiography was suggested in the fracture liaison services (FLS). Osteoporotic VCFs usually occur at the thoracolumbar junction, and their diagnosis by plain radiography is sometimes difficult because of the superimposed pulmonary hyperlucency and the progressive bone mineral density reduction related to aging. Other imaging modalities, such as computed tomography (CT) and magnetic resonance imaging (MRI), may be required for accurate diagnosis of VCFs, as well as for the evaluation of fracture extent and bone healing (2, 3). However, these advanced imaging modalities are not the standard first-line imaging in healthcare facilities because of several drawbacks, including long waiting lists due to the growing need caused by the large population with osteoporosis, higher costs, the longer examination time and contraindications for MRI (like claustrophobia and pacemakers), and the larger radiation dose of CT.

In addition, close monitoring of VCF healing is essential and has a profound clinical and socio-economic impact. Patients may be relieved of severe back pain and return to normal activity after adequate VCF healing under appropriate treatment, such as medication, brace protection, vertebroplasty, or spine alignment correction with pedicle screw placement. Contrarily, VCFs could lead to disability, morbidity, and mortality, if untreated. Radiographic evidence of VCF healing encompasses blurring of the fracture line, callus formation, and bridging of the cortical gap (4). Plain radiography remains the most popular tool in VCF healing assessment because of its low cost and convenience; however, its limitations and the weak correlation between radiographic bone healing conditions and biomechanical clinical symptoms often result in decision-making challenges for surgeons regarding the necessity of further invasive treatment.

Tomosynthesis has been available since 2008, and its application has been evaluated in several fields, such as chest radiography (5), mammography (6), musculoskeletal radiography, and fracture healing assessment (7). It was also found to be a useful tool for the evaluation of the spine, particularly the thoracolumbar area, where the overlaying anatomy is accentuated. Studies investigating the utility of tomosynthesis in assessing spinal injury in ankylosing spondylitis (8) and for predicting prevalent VCFs in multiple myeloma (9) have shown promising results. The Taiwan TomoDR tomosynthesis imaging system is a new X-ray imaging modality developed by the Institute of Nuclear Energy Research (10). A series of low-dose exposure projection radiographs can be acquired over a limited angular range with the reconstruction of a large amount of information to provide detailed image information at each depth.

The purpose of this study was to evaluate the performance of TomoDR by assessing the spinal anatomy in patients with new identified VCFs in our institute, including accuracy of diagnosis, the visibility of anatomic details of fractured and adjacent levels, and bone healing assessment at the 3-month follow-up period in the FLS program.

## Patients and methods

### Patient information

**FIGURE 1:**
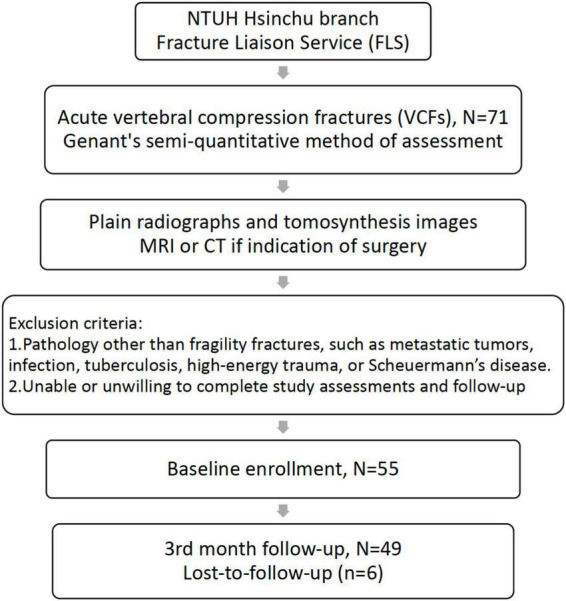
Study flow chart. Patients were excluded if (1) VCFs were related to other pathologies and (2) they were unable or unwilling to complete study assessments and follow-ups. VCFs, vertebral compression fractures.

Patients enrolled in the FLS program were required to be older than 50 years and meet one of the following three conditions: (1) new hip fracture; (2) new identified VCFs; or (3) clinical VCFs in the National Taiwan University Hsin-Chu Hospital. This study prospectively recruited patients having acute back pain with new identified VCFs between August 2018 and August 2020 from the FLS database. All patients underwent thoracolumbar plain radiography and tomosynthesis within one week after enrollment and at a 3-month follow-up period. Patients with a possible surgical indication underwent MRI or CT within the acute phase (4 weeks). Patients with VCFs because of metastatic tumors, infection, tuberculosis, high-energy trauma, Scheuermann’s disease, or those with inability or unwillingness to complete study assessments and follow-up were excluded. The study was conducted according to the guidelines of the Declaration of Helsinki and approved by the Institutional Review Board of the National Taiwan University Hsin-Chu Hospital. The IRB was approved on 28 February 2018 (IRB number is 107-028-F), and informed consent was obtained from all patients. Figure 1 shows the study flowchart.

### Image acquisition

**FIGURE 2:**
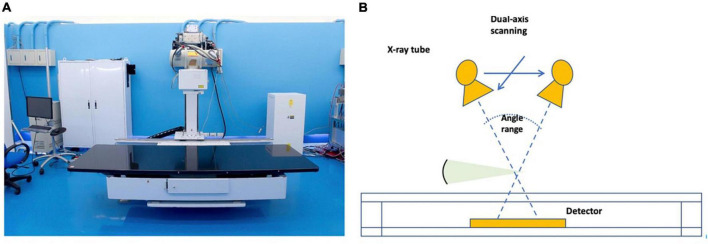
**(A)** Taiwan TomoDR X-ray tomosynthesis system; **(B)** schematic illustration of the range of motion of TomoDR.

Lateral thoracolumbar plain radiographs, tomosynthesis, CT, and MRI images were obtained from the same institute. Patients were placed in the right lateral recumbent position during the tomosynthesis examination. TomoDR (Figure 2), a prototype accounted with dual-axis scanning geometry in two perpendicular directions, is a tomosynthesis imaging system (10). A digital flat-panel image receptor (Model: PaxScan 4343CB, Varian Medical Systems, United States) and a medical X-ray source (Model: SG-1096, Varian Medical Systems, United States) were assembled to build the system. This digital tomosynthesis system can provide repeated accuracy of positioning within 50 μm and meets all requirements of position repeatability of the motion mechanism of the dual-axis. System configurations have a 1,100 mm source-to-image receptor distance. The X-ray source moves along the head-foot or left-right axis during image acquisition, and the patient and image receptors remain immobile. A 3,072 × 3,072-pixel matrix flat-panel detector with a pixel size of 0.139 mm × 0.139 mm assembled the image receptor, and a 2 × 2-pixel binning of the image receptor was used to enable a higher number of frames captured per second. The X-ray source position moves from −300 mm (−15.25°) to +300 mm (+15.25°) at 10-mm increments in the head-foot and left-right axis direction in this clinical spine scanning protocol. The number of projection images in each direction was 61, and the scanning protocol of the spine tomosynthesis imaging was 80 kV and 1.6 mA for each projection. A total of 122 projection images were reconstructed using a maximum-likelihood expectation-maximization algorithm in a 1,024 × 1,024 × 200-pixel matrix with a voxel size of 0.5 mm × 0.5 mm × 1 mm (11) in this spine reconstruction image protocol.

### Accuracy of vertebral compression fracture diagnosis

All images were evaluated on the same spine length. Three experts (one radiologist with 20 years of experience and two orthopedic surgeons with 5 and 9 years of experience, respectively) who were blinded to the clinical symptoms of the patient reviewed the plain radiographs and reconstructed TomoDR section images independently. Each reader evaluated the plain radiographs first, followed by the TomoDR images of each patient in random order. The complete series of TomoDR section images were accessible during the evaluation and displayed to the experts with a calibrated medical-grade monitor. The experts were allowed to pan and zoom for window width and level adjustment. The levels of VCF (decreased vertebral body height of more than 20%) were recorded, which was considered acute compression fracture if signs of cortical breaking or impaction of trabeculae were present and chronic if the signs were absent. The diagnostic accuracy of VCFs between plain radiographs and TomoDR images was evaluated using spine MRI as the gold standard in the patients who underwent spine MRI in the acute phase. Vertebral collapse with bone marrow edema on short tau inversion recovery MR images was considered acute VCF, while vertebral collapse without bone marrow edema was considered chronic VCF. The MR images were evaluated in consensus by the three experts blinded to the clinical symptoms of the patients and the results of the other imaging studies. Any disagreements between the readers were solved by discussion to reach a consensus. Considering the slightly different coverage of different imaging modalities, only T10–L5 vertebrae (eight levels) were evaluated.

### Image quality analysis and vertebral compression fractures healing assessment

Comparison between lateral thoracolumbar radiographs and TomoDR images was made based on several image quality criteria. A vertebral level was defined as evaluable when more than three out of the following five structures could be clearly seen: upper and lower endplates, anterior and posterior vertebral edges, and cancellous bone of the vertebral body. Several anatomic structures of the target levels (collapsed levels) including upper and lower endplates, anterior and posterior vertebral edges, cancellous bone of the vertebral body, pedicles and neuroforamina, and articular and spinous processes were evaluated for their visibility and clarity. Visual grading characteristic (VGC) analysis was used to determine the difference in image quality using a five-step rating scale, as follows: (1) unacceptable or indecisive; (2) poor confidence; (3) good confidence; (4) high confidence; and (5) complete confidence. The three readers were experienced in reading spine radiographs and CT, and after test reading of a few images, they reached a consensus that if the image quality was almost as good as CT, it was rated 5, and if the image quality was like standard radiographs, it was rated 3. The visibility of the fracture lines and their extents were also recorded, including upper or lower endplate involvement, anterior and posterior border involvement, intravertebral fracture, presence of retropulsion, and posterior element involvement.

The 3-month-follow-up images were focused on VCF healing assessment. Images of a sufficiently high quality that allowed evaluation of the presence of callus formation (such as cortical bone bridging) or absence of union (such as visible fracture line or vacuum phenomenon) scored 1 point in cases with confidence. Those that failed to provide adequate image quality of the target level for assessing bone healing scored 0 in cases with no confidence.

### Statistical analysis

The VGC points were plotted to produce a receiver operating characteristic (ROC) curve, and the area under the curve was used as a measure for comparing the lateral thoracolumbar radiographs and tomosynthesis images. Pearson’s Chi-square test was used to compare the results through sensitivity, specificity, positive predictive value, negative predictive value, and accuracy. The significance level for all analyses was set to 0.05, and IBM SPSS Statistics v.22.0 (IBM Corporation, Armonk, NY, United States) was used to perform statistical analyses.

## Results

### Patient characteristics

This study included 49 patients with a mean age of 78 (range, 51–94) years, and the overall follow-up rate was 89.1% (49/55). The average schedule time of thoracolumbar plain radiography and tomosynthesis is 4 (range, 1–7) days, and average schedule time of MRI or CT is 24 (range, 14–35) days. The costs of a single lateral thoracolumbar plain radiograph, tomosynthesis, CT, and MRI are approximately $10, $30, $165, and $282, respectively, in our hospital. All examination fees were sponsored by the Ministry of Science and Technology of the Republic of China (MOST) grants. A total of 21 patients underwent MRI, and 6 patients underwent CT examination. The patients’ demographic and clinical data and response to the visual analog scale are shown in Table 1.

**TABLE 1:** Patient demographics and clinical status of vertebral compression fractures in 49 patients.

Variable	Values no. or median
Sex	
Men	10 (20.4%)
Women	39 (79.6%)
Age (year)	78 (51–94)
Body_height (cm)	152 (136–178)
Body weight (kg)	54.5 (38–89)
Pre op pain score, VAS (0–10)	
None (0)	0
Mild (1–3)	0
Moderate (4–6)	18 (36.7%)
Severe (7–10)	31 (63.3%)

Values are median and interquartile range in parentheses or number with percentage in parentheses.

VAS, visual analog scale.

### Accuracy of vertebral compression fracture diagnosis

Tomosynthesis identified 90 levels of VCF in 49 patients, while plain radiography revealed only 87.8% (79/90) of them. There were 44.9% (22/49) patients with neglected chronic VCFs as seen on tomosynthesis. In the 21 patients who underwent MRI, there were 25 acute VCFs, 19 chronic VCFs, and 124 normal vertebrae among the 168 evaluated levels (Table 2). For diagnosis of VCFs, the performance of plain radiographs was poorer than that of tomosynthesis images (plain radiographs: sensitivity 84%, specificity 93.5%, false positive rate 6.5%, and false negative rate 16%; tomosynthesis: sensitivity 93.2%, specificity 100%, false positive rate 0%, and false negative rate 6.8%). Kappa coefficient is 0.704 (*p* < 0.001) between plain radiographs and MRI and is 0.956 (*p* < 0.001) between tomosynthesis and MRI. For diagnosis of acute VCFs, the performance of plain radiographs was poorer than that of tomosynthesis images (plain radiographs: sensitivity 84%, specificity 93.7%, false positive rate 6.3%, and false negative rate 16%; tomosynthesis: sensitivity 88%, specificity 100%, false positive rate 0%, and false negative rate 12%). The findings were similar for the diagnosis of chronic VCFs (plain radiographs: sensitivity 52.6%, specificity 96.7%, false positive rate 3.3%, and false negative rate 47.4%; tomosynthesis: sensitivity 100%, specificity 100%, false positive rate 0%, and false negative rate 0%).

**TABLE 2:** Comparison of the performance of X-ray and tomosynthesis in diagnosis with MRI.

		Acute	Chronic	Normal	Total
MRI		25	19	124	168
**X-ray**	Acute	21	6	3	30
	Chronic	0	10	5	15
	Normal	4	3	116	123

**Tomo**	Acute	22	0	0	22
	Chronic	0	19	0	19
	Normal	3	0	124	127

Tomo, tomosynthesis; MRI, magnetic resonance imaging.

### Image quality analysis and healing assessment

**FIGURE 3:**
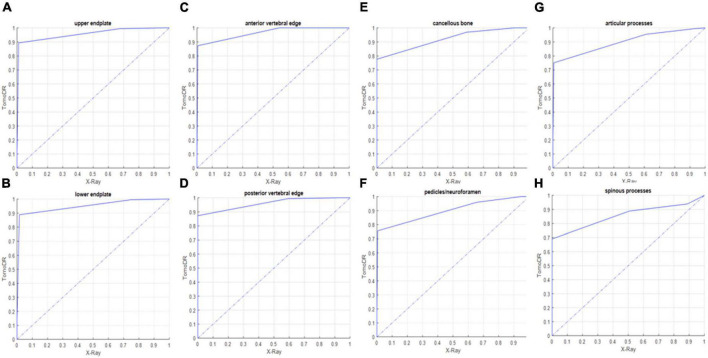
Receiver operating characteristic (ROC) curve between TomoDR and radiography in the assessment of the **(A)** upper and **(B)** lower endplates, **(C)** anterior and **(D)** posterior vertebral edges, **(E)** cancellous bone, **(F)** pedicles/neuroforamina, **(G)** articular, and **(H)** spinous processes of the target levels (fractured levels) using visual grading characteristic (VGC). TomoDR shows better performance in the evaluation of all the structures.

**FIGURE 4:**
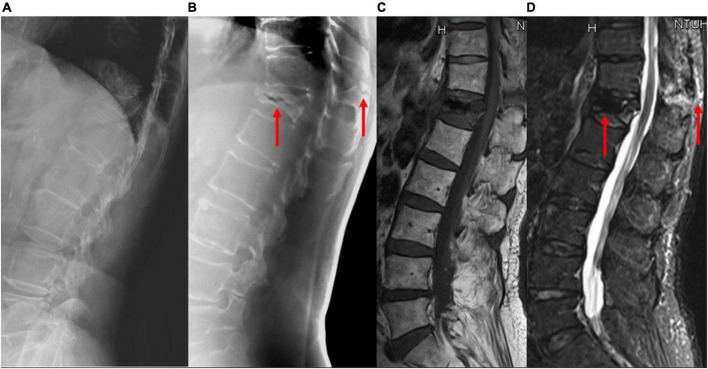
A patient with acute VCF at the T-L junction. The bony details of the fracture and the adjacent vertebral levels are ruined by the abrupt density transition from the lungs to the abdomen and the decreased bone mineral density on **(A)** lateral radiographs; **(B)** tomosynthesis; **(C)** T1-weighted MRI, and **(D)** short tau inversion recovery MRI images clearly show the fracture involving the anterior and posterior vertebral edges and the spinous process (arrows). VCF, vertebral compression fracture; T-L, thoracolumbar; MRI, magnetic resonance imaging.

**FIGURE 5:**
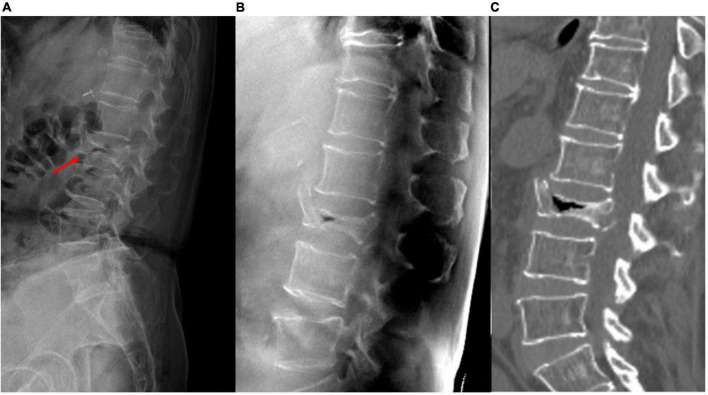
A patient with acute L2 vertebral compression fracture (VCF). **(A)** Plain radiograph shows bowel gas superimposed on the spine, and the fracture lines of the collapsed vertebra cannot be clearly seen. **(B)** Tomosynthesis and **(C)** CT both clearly show the extent of fractures and the air-filled cleft.

The results of the image quality analysis are shown in Table 3. TomoDR images showed all the subsites more clearly than the radiographs. The area under the VGC values were 0.957 for the upper endplate, 0.949 for the lower endplate, 0.964 for the anterior vertebral edge, 0.96 for the posterior vertebral edge, 0.961 for the cancellous bone, 0.899 for the pedicles/neuroforamina, 0.9 for the articular processes, and 0.857 for the spinous process (Figure 3). The fracture extent was more clearly visible on TomoDR images than on plain radiographs (Figures 4, 5). For the fracture line assessment, TomoDR image scores were higher than those of plain radiographs by 1.16 times for the upper endplate, 2.04 times for the lower endplate, 1.36 times for the anterior vertebral edge, 1.56 times for the posterior vertebral edge, and 1.36 times for the intravertebral cancellous bone. No significant difference was seen between the two image modalities for evaluable vertebral body levels.

**TABLE 3:** The results of the image quality score analysis.

Anatomy	Upper	Lower	Anterior	Posterior	Cancellous	Pedicles/	Articular	Spinous
	endplate	endplate	vertebral edge	vertebra edge	bone	Neuroforamina	processes	processes
Imaging quality	X ray							
A (5)	0	0	0	0	0	0	0	0
B (4)	2	3	1	0	0	1	1	0
C (3)	131	144	105	117	116	128	119	100
D (2)	54	6	69	63	61	57	63	75
E (1)	10	17	22	17	20	11	14	22
Total	197	197	197	197	197	197	197	197

Imaging quality	TomoDr							
A (5)	26	30	24	16	0	36	42	39
B (4)	150	145	148	156	153	113	106	97
C (3)	20	21	25	24	38	40	40	39
D (2)	1	1	0	1	6	8	8	10
E (1)	0	0	0	0	0	0	1	12
Total	197	197	197	197	197	197	197	197

**FIGURE 6:**
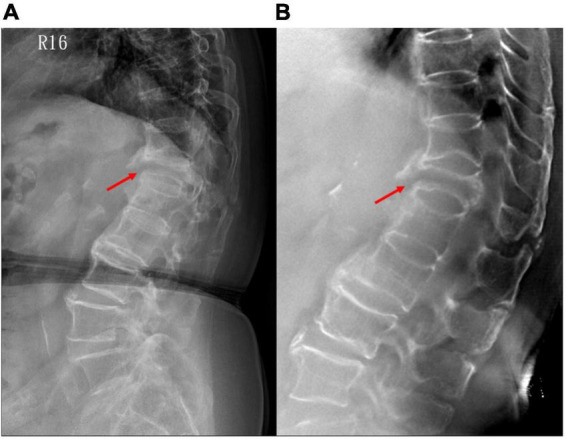
A patient with non-union of VCF on the 3-month follow-up. **(A)** Plain radiograph shows non-union with a gap at the anterior vertebral edge (arrow); while **(B)** tomosynthesis more clearly demonstrates both anterior and posterior vertebral edge involvement. VCF, vertebral compression fracture.

**FIGURE 7:**
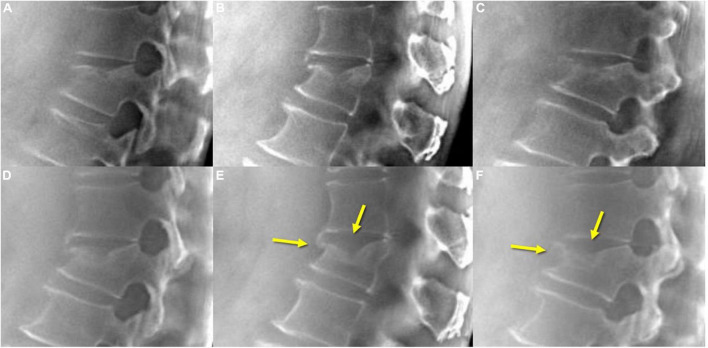
TomoDR in acute VCF **(A–C)** shows fracture lines extending to the upper endplate and anterior vertebral edge, while on the 3-month follow-up **(D–F)** images, a fusion of the fracture lines is clearly demonstrated (arrows). VCF, vertebral compression fracture.

Furthermore, TomoDR images scored 3.3 times higher than plain radiographs on the fracture healing assessment at the 3-month-follow-up period (TomoDR score: 43 vs. radiograph score: 13). TomoDR demonstrates superior detection of non-union of VCFs in the follow-up stage (Figure 6) and is effective for fracture healing process assessment (Figure 7).

## Discussion

Early diagnosis of VCFs is challenging because they may be asymptomatic or only cause mild pain; this makes it a highly underdiagnosed disease. VCFs are associated with a significant loss of independence, morbidity, and mortality, and they incur high societal costs. The incidence of VCFs increases with the aging of the population and there is a lack of routine radiographic detection in the clinical pathway. Current diagnostic tools still rely on conventional methods, such as medical history, physical examination, and lateral thoracolumbar radiographs. Physical examination findings such as exaggerated kyphosis (12) or height loss of over 4 cm may imply the presence of radiographic VCFs (13). Lateral thoracolumbar radiography continues to be the most popular modality due to its price and availability (14). Other spinal imaging modalities, such as CT, MRI, and radionuclide bone scanning, are reserved for those who need evaluation of the sharpness of fracture lines or differential diagnosis between osteoporotic and pathologic fractures (15).

There were 44.9% (22/49) patients with neglected chronic VCFs in our study. The early detection of VCFs is paramount in the effort to decrease secondary osteoporotic fractures. Due to limited existing manpower and resources, it is challenging to detect VCFs in real world practice. Computer-driven solutions integrated with deep convolutional neural networks have been used for screening, detection, and localization of VCFs (16). MRI is considered the state of the art in investigating VCFs, and some VCFs, particularly acute, chronic, and healing process, are only visible in MRI. Plain radiography is known to miss numerous subtle VCFs, and CT exhibits an AUC of only 0.85 in differentiated acute and chronic VCFs compared to MRI (17). A better screening tool that can improve accuracy and efficiency in the diagnosis of VCFs is critically needed; thus, the cost-effectiveness and accuracy of tomosynthesis make it a valuable tool. In this prospective study, the performance of tomosynthesis for VCF diagnosis assessment was evaluated by comparing it with that of plain radiography in FLS. Tomosynthesis images increased the diagnostic accuracy of VCFs by 12.2% compared to plain radiography. Moreover, we used MRI, which is considered the standard reference for diagnosing VCFs, to confirm the exact levels of acute VCFs and the fracture extent. Kappa coefficient is 0.704 (*p* < 0.001) between plain radiographs and MRI and is 0.956 (*p* < 0.001) between tomosynthesis and MRI in diagnosis of VCFs. The results indicate tomosynthesis has perfect agreement with MRI, while the agreement between plain radiograph and MRI has substantial agreement. Tomosynthesis has the potential providing almost equal diagnosis accuracy of VCFs to MRI.

With the use of different reconstruction protocols, clinicians can choose to focus on specific bone structures, such as the vertebral body, pedicle, or facet joints/neuroforamina. Therefore, tomosynthesis may be an effective tool for accurately diagnosing VCFs, which often occur in the thoracolumbar junction, as the quality of plain radiographs is commonly impaired by anatomic noises from the heart or lungs and the age-related bone mineral density decrease (Figure 4). Tomosynthesis has the advantage of multiple slices, which can provide more image information than plain radiographs. Further, with multislice images, the difficulty in image interpretation due to overlapping anatomical structures or bowel gas can be avoided (Figure 5).

The results of this study indicated that VCFs in patients with osteoporosis are better detected with tomosynthesis than with plain radiographs. The mean difference in the image quality scores and the user preference were significantly in favor of tomosynthesis, suggesting that this imaging modality can assist clinicians to correctly diagnose VCFs. Tomosynthesis images allowed clinicians to see the extent of the fracture line more clearly, to determine whether it affects the upper, lower, anterior, and posterior borders, and the intravertebral trabeculae, and to evaluate no fewer vertebrae than on lateral thoracolumbar radiographs. We did not include CT for every patient in the protocol due to radiation dose considerations; however, based on the CT images of a few patients in our study, tomosynthesis seemed to provide imaging quality similar to that of CT.

Another advantage of tomosynthesis is the low-dose radiation. The average effective dose for thoracolumbar radiographs was reported to be 1.0 mSv, ranging from 0.6 to 1.4 mSv, and that for CT of the spine was 6 mSv, ranging from 1.5 to 10 mSv (18). The radiation dose for tomosynthesis in this study was 12–17% of the standard dose of CT of the spine (19).

Assessment of the vertebral bone healing condition is important for surgeons to determine whether the patient needs further surgical treatment and is mostly based on experts’ opinion without general agreement on radiographic criteria or clinical symptoms (20). CT or MRI may be used, but there is a wide variety in both radiographic criteria and clinical symptoms. In the present study, tomosynthesis had superior performance in VCF healing assessment compared to radiography and may yield more useful information regarding the next steps in practice (Figure 6). The performance of tomosynthesis for VCF healing assessment scored 3.3 times more for VCF healing assessment than did plain radiographs. Tomosynthesis may solve the weakness of plain radiographs in evaluation of bone healing condition and assist surgeons in more precise decision-making and patient management. Additionally, it is difficult to differentiate acute from chronic VCFs on plain radiographs, although radiographic signs such as cortical breaking and impaction of trabeculae suggest an acute fracture. Tomosynthesis can show the fracture line, cortical buckling, and trabecular impaction of acute VCFs and the callus formation, remodeled appearance, and non-union of chronic VCFs more clearly (Figure 7).

The disadvantages of tomosynthesis include the following. First, it can only provide multislice images in the sagittal plane, which is the most common diagnostic plane for VCFs. Thus, fractures that are more readily seen on AP projection, such as lateral process fractures, might not be easily detected. Second, the evaluation of VCFs is more difficult in patients with degenerative scoliosis, in particular the details of the adjacent levels. However, tomosynthesis is still superior to conventional radiography for this condition. Third, the sharpness of tomosynthesis images decreases as the distance from the focus target vertebrae or structure increases. The stated slice thickness of tomosynthesis images is actually the slice increment, which is different from that of CT. The interpreters should acknowledge the differences.

In studies with radiologic readings, the number of readers is critical to avoid personal bias and allow different opinions. The semiquantitative scoring, the number of readers, and the supportive clinical and imaging data, such as clinical course, CT, or MRI, are the strengths of this prospective study.

There are also some limitations of this study. First, the number of patients and examinations were relatively small; however, it meets the standard for demonstrating a statistically significant difference. Second, not all patients with acute VCFs underwent CT or MRI due to the consideration of unnecessary radiation exposure and indications. CT should be better than MRI because radiography, tomosynthesis, and CT are all X-ray imaging products. Despite these limitations, our results are valuable as they support the use of TomoDR as a new screening tool for VCFs owing to its diagnostic accuracy compared with plain radiography. Nonetheless, future studies with larger numbers of patients and longer follow-up periods are required.

## Conclusion

In conclusion, tomosynthesis, expected to be an alternative modality for CT or MRI, is a promising screening tool for the diagnosis of VCFs in patients with osteoporosis and for monitoring of the fracture healing status at a similar radiation dose and cost as those of plain radiography. This modality may improve the accuracy and efficiency in the diagnosis and treatment of VCFs and may have an impact on the medical economics of the aging population.

## Data availability statement

The original contributions presented in this study are included in the article/supplementary material, further inquiries can be directed to the corresponding author.

## Ethics statement

The study was conducted according to the guidelines of the Declaration of Helsinki and approved by the Institutional Review Board of National Taiwan University Hsin-Chu Hospital (IRB number: 107-028-F). The patients/participants provided their written informed consent to participate in this study.

## Author contributions

H-YC, S-PT, and C-WC: conceptualization. S-PT and C-YL: formal analysis and funding acquisition. H-YC, C-YL, and Y-FC: methodology. TW, S-PT, and Y-FW: project administration. H-YC, C-WC, and Y-FC: resources. S-PT: software. TW, T-HW, and Y-FC: supervision. H-YC and Y-FC: validation and writing—original draft. H-YC, T-HW, and Y-FC: writing—review and editing. All authors contributed to the article and approved the submitted version.
